# A Neonatal Intensive Care Unit’s Experience with Implementing an In-Situ Simulation and Debriefing Patient Safety Program in the Setting of a Quality Improvement Collaborative

**DOI:** 10.3390/children7110202

**Published:** 2020-10-29

**Authors:** Mary Eckels, Terry Zeilinger, Henry C. Lee, Janine Bergin, Louis P. Halamek, Nicole Yamada, Janene Fuerch, Ritu Chitkara, Jenny Quinn

**Affiliations:** 1 Maternal Newborn Services, St. Jude Medical Center, Fullerton, CA 92835, USA; mary.eckels@providence.org (M.E.); terry.zeilinger@providence.org (T.Z.); 2 Neonatal Intensive Care Unit, Lucile Packard Children’s Hospital, Stanford, CA 94305, USA; hclee@stanford.edu (H.C.L.); halamek@stanford.edu (L.P.H.); nkyamada@stanford.edu (N.Y.); jfuerch@stanford.edu (J.F.); chitkara@stanford.edu (R.C.); 3 California Perinatal Quality Care Collaborative (CPQCC), Stanford, CA 94305, USA; jmbergin@stanford.edu; 4 Center for Advanced Pediatric and Perinatal Education (CAPE), Stanford, CA 94305, USA; 5 NeoQIP (Neonatal Quality Improvement Performance) LLC, Martinez, CA 94553, USA

**Keywords:** neonatal simulation, simulation, debriefing, quality improvement, collaborative, neonatal intensive care unit, in-situ simulation, patient safety

## Abstract

Extensive neonatal resuscitation is a high acuity, low-frequency event accounting for approximately 1% of births. Neonatal resuscitation requires an interprofessional healthcare team to communicate and carry out tasks efficiently and effectively in a high adrenaline state. Implementing a neonatal patient safety simulation and debriefing program can help teams improve the behavioral, cognitive, and technical skills necessary to reduce morbidity and mortality. In *Simulating Success*, a 15-month quality improvement (QI) project, the Center for Advanced Pediatric and Perinatal Education (CAPE) and California Perinatal Quality Care Collaborative (CPQCC) provided outreach and training on neonatal simulation and debriefing fundamentals to individual teams, including community hospital settings, and assisted in implementing a sustainable program at each site. The primary Aim was to conduct two simulations a month, with a goal of 80% neonatal intensive care unit (NICU) staff participation in two simulations during the implementation phase. While the primary Aim was not achieved, in-situ simulations led to the identification of latent safety threats and improvement in system processes. This paper describes one unit’s QI collaborative experience implementing an in-situ neonatal simulation and debriefing program.

## 1. Introduction

During the last couple of decades, literature suggests there is ongoing patient morbidity and mortality associated with avoidable harm and medical errors, including errors that can be prevented with improved communication and systems design [1,2]. Healthcare is a complex, dynamic process involving many disciplines collaborating in an effort to provide optimal care. In the U.S., most births do not require advanced resuscitation (i.e., positive pressure ventilation, chest compressions, or medication administration); however, about 10% of infants born do require some resuscitative efforts [3]. Approximately one percent will require extensive resuscitation following birth, including chest compressions and medication administration [3]. Because extensive neonatal resuscitation is infrequent, resuscitation can be compromised because of cognitive and task overload, poor communication, and lack of teamwork. In-situ simulation provides a realistic context and opportunity for staff to practice problem-solving, decision-making, teamwork, and critical thinking skills in the actual delivery care setting [4]. Additionally, interprofessional in-situ simulation is a useful tool to help promote quality in neonatal care [5] and has been shown to identify latent safety threats (LSTs) [6]. This is in contrast with simulations conducted in a simulation center, in which there may be incomplete teams or healthcare professionals, who may not necessarily work with each other [6].

LSTs are errors in design, organization, training, or maintenance that can lead to medical errors [7]. LSTs are categorized into medication errors, equipment malfunction/misuse, inadequate teamwork, and other findings [8]. The relatively low frequency of neonatal resuscitation (especially extensive resuscitative interventions) creates an opportunity for LSTs to occur. In a retrospective study examining LSTs in a pediatric intensive care unit through an in-situ simulation program, 41% of the LSTs that were identified had the potential to cause harm. Category analysis showed that the majority of these LSTs were due to either a lack of resources (36%) or lack of education and training (27%) [9]. In-situ simulation training can help identify LSTs in the real-life clinical setting where maternal deliveries and neonatal resuscitations actually occur. As a result, this type of training can test systems and uncover gaps in processes that may delay timely care. In-situ simulations can assist in detecting LSTs and minimize the risk of a serious safety event [7]. For example, the simulation of a placental abruption followed by neonatal hypovolemia and perinatal depression can test the system and processes that are in place to include the blood bank, laboratory, respiratory therapy, and the maternal and neonatal teams. One metric to evaluate the effect of this simulated scenario is to measure the time required to obtain emergency O-negative blood from the hospital blood bank. Timely recognition and mitigation of LSTs are essential to reducing preventable harm during neonatal resuscitation. 

Quality improvement (QI) collaboratives have grown increasingly popular. As such, there has been an exponential rise in perinatal and neonatal collaboratives across the United States, including California, Massachusetts, Florida, Tennessee, Ohio, New York, and Oregon. Since the inception of collaboratives in many states and regions throughout the United States, QI projects have covered various topics such as antenatal corticosteroid administration, antibiotic stewardship, and neonatal abstinence syndrome [10]. QI collaboratives create an environment for shared learning among healthcare organizations and professionals. In this shared learning environment, QI collaborative participants are supported by a faculty of experts who identify better practices and help facilitate implementation strategies to improve care [11]. Through monthly webinars and face-to-face interactive sessions, shared learning among different healthcare organizations and professionals occurs. Teams come together to learn, apply, and share not only their approaches to QI but also their data, successes, and barriers to improved performance [11].

We describe our unit’s experience participating in *Simulating Success*, a QI collaborative hosted by California Perinatal Quality Care Collaborative (CPQCC) and the Center for Advanced Pediatric and Perinatal Education (CAPE) through Stanford. CPQCC, established in 1997, is a non-profit quality improvement organization dedicated to improving the delivery of care and outcomes for California’s mothers and vulnerable infants. CAPE, the world’s first simulation center founded in 2002, is dedicated to neonatal and pediatric simulation-based training and research. The QI collaborative focused on implementing an in-situ neonatal simulation and debriefing program. Although the collaborative had common principles of implementation across centers, each individual center had their own Aims and process for implementation. We were provided ongoing assistance throughout the collaborative with a QI specialist that helped our team create Aim statements, identify metrics to assess progress in the collaborative, and review the concepts and assist core team members with the development of plan-do-study-act (PDSA) cycles. 

## 2. Materials and Methods

### 2.1. Training and Preparation

Our Neonatal Intensive Care Unit (NICU) is a 14-bed, community Level III NICU located in Southern California. In 2019, there were 2354 deliveries and 211 NICU admissions with an average daily census (ADC) of six. Training and preparation to implement an in-situ neonatal simulation program consisted of identifying three core team members from our unit: a neonatologist/NICU Medical Director; a clinical nurse IV (the highest position on the clinical ladder for a staff nurse) that works the day shift; and a clinical coordinator (charge nurse) for the NICU on the night shift.

**Figure 1:**
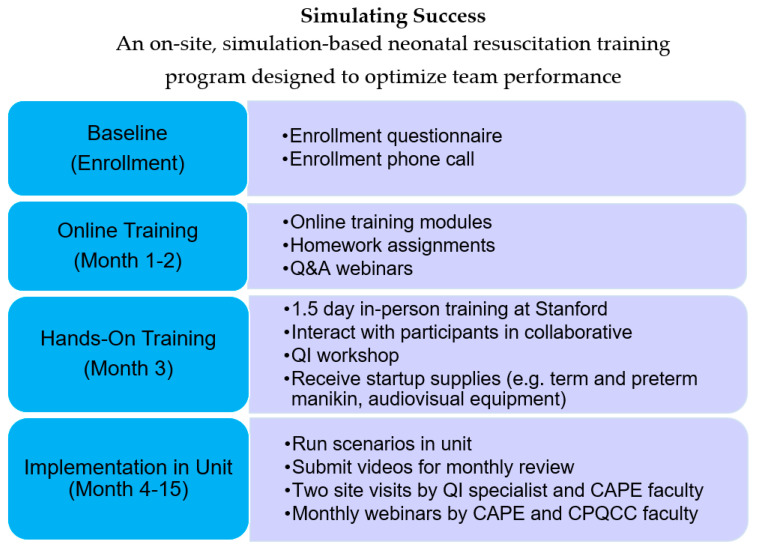
*Simulating Success* Project Timeline.

Participation in *Simulating Success* lasted 15-months, including three months of pre-implementation work prior to the 12-month active implementation phase (Figure 1). During the three-month pre-implementation phase, from July 2018 to September 2018, the core team members viewed nine online modules developed by CAPE. This series of modules covered a broad scope of simulation and debriefing techniques developed by the CAPE faculty. Homework assignments were included with each module; these involved the development of simulated clinical scenarios that our NICU experienced on a daily basis (high-risk, high volume) and scenarios that were not as frequently encountered (high-risk, low volume). During this same timeframe, monthly webinars were conducted with CAPE, CPQCC faculty, and a QI specialist in preparation for the simulation and debriefing course conducted at CAPE. In October 2018, our three-person team attended a 1.5-day intensive CAPE simulation and debriefing course and had the opportunity to engage with CAPE and CPQCC faculty and perform simulated clinical scenario followed by debriefings. During this intensive 1.5-day training, these same core team members also had the opportunity to debrief another NICU team’s simulated clinical scenario and have our debriefing critiqued by the CAPE team, essentially a debriefing of our debriefing. During the months of November and December 2018, our team solidified simulation team members, developed Aim statements for the project, procured simulation materials and supplies, and discussed space allocation for simulations and debriefings. 

Our core team was provided ongoing assistance throughout the collaborative with a QI specialist that helped our team create Aim statements, identify metrics to assess progress in the collaborative, and review the concepts and assist core team members with the development of plan-do-study-act (PDSA) cycles. CPQCC and CAPE had two patient outcome-related Aims for the overall collaborative. As a small NICU, we were concerned that we would not have enough patient data to demonstrate improved patient care outcomes. Without accurate and timely data evaluation and feedback to staff, we were concerned that this could impact short and long-term staff buy-in. Our NICU’s overall goal and reason to participate in this collaborative were to implement a multidisciplinary in-situ simulation program to improve team performance and debriefing skills. Therefore, our primary Aim statement indicated that the training team would conduct two video-recorded in-situ simulations and debriefings in the NICU every month from January 2019 through December 2019. Our second Aim statement declared that 80% of NICU staff would participate in two in-situ simulations and debriefings on an annual basis by December 2019. The QI specialist also worked with our team and provided suggestions on how to implement the simulation and debriefing program.

### 2.2. In-Situ Simulation Implementation Process

Our team decided to implement an in-situ simulation and debriefing program in which simulations and debriefings are conducted in labor and delivery or the NICU. Leadership in nursing and respiratory therapy supported and promoted the project through budgetary and personnel resources. A presentation explaining *Simulating Success* and the collaborative’s goals and objectives were presented at a Maternal Newborn Services Division and Respiratory Services Department meeting. Additional communications occurred via email and during shift change reports. 

Initially, the two nurse (RN) members of the core team set up and facilitated the first in-situ simulations utilizing simulations previously developed at CAPE. The neonatologist/medical director and respiratory care professional (RCP) members assisted by fulfilling different roles necessary to make the simulated scenarios realistic. In-situ simulations were held approximately two times per month, starting in January 2019. After a few months of becoming familiar with the simulation-based and audiovisual technologies and gaining practice with debriefing, the core team presented an in-person, interactive in-service training session based on CAPE’s simulation and debriefing principles. This learning session was open to NICU RCP and RN clinical coordinators (charge nurses) and the goal was to integrate the CAPE debriefing methodology into routine simulations and utilize the debriefing techniques with real-life events. The in-service was also designed to incorporate other staff as part of succession planning for core team leaders. Training additional staff allows for the sustainability and expansion of in-situ simulations and debriefings at our institution.

Each month during the one-year implementation phase, January 2019 through December 2019, we submitted our in-situ simulation and debriefing videos to the team at CAPE for their review and critique. Additionally, CAPE and CPQCC faculty hosted monthly webinars attended by our core team members and another NICU participating in Group 2. In June 2019, Group 2 (*n* = 2) combined with Group 3 (*n* = 3) for the monthly webinars. The two groups were combined to enhance the shared learning environment and unit experiences. During these webinars, CAPE faculty offered suggestions on scenario design and implementation as well as techniques for enhancing realism. In addition, the CAPE team critiqued our recorded debriefings. Participating NICUs were also afforded the opportunity to share the barriers to implementation and strategies for success that each experienced. 

All subjects gave their informed consent for inclusion before they participated in the study. The study was conducted in accordance with the Declaration of Helsinki, and the protocol was approved by the Ethics Committee of Stanford (Project identification 38601).

## 3. Results

Aim 1: Between January 2019 and December 2019, the team conducted 15 in-situ simulations (goal 24) in our Maternal and Newborn Services Division, averaging roughly one simulation per month. The main challenge to conducting in-situ simulations was a persistently high census in the NICU that limited the ability of staff to participate without potentially compromising the care of actual patients. 

Aim 2: In 2019, 70% of our staff (goal 80%) attended two or more simulated scenarios. Seven of the staff (26%) attended only one simulation during that time. We fell short of our goal primarily due to medical leave or per diem schedules.

### Latent Safety Threats

**Figure 2:**
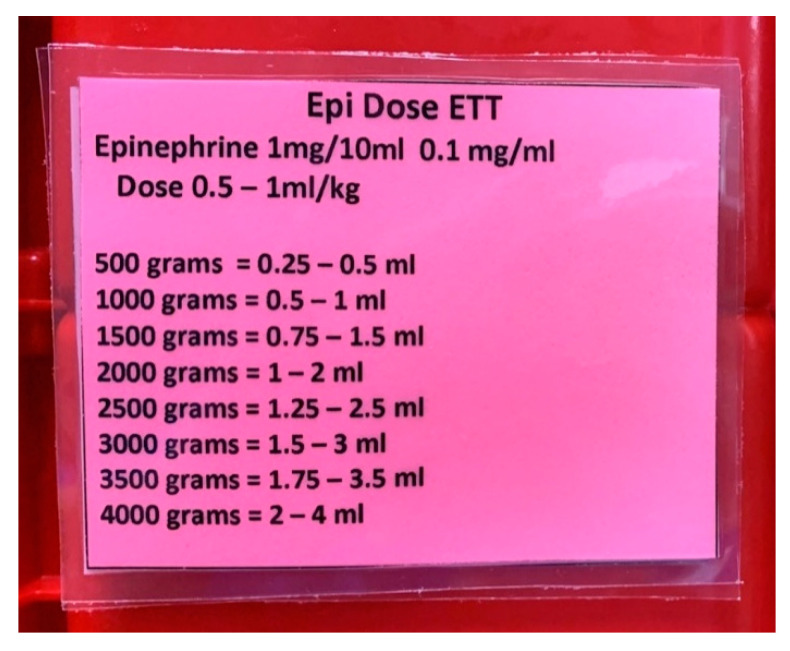
Epinephrine Administration via Endotracheal Tube Dosing Card Inside the Tackle Box.

Participation in *Simulating Success* revealed a total of four LSTs in our unit. The first LST identified involved the administration of epinephrine. Although the team was able to ascertain and give the correct dose of epinephrine utilizing the standard preprinted, weight-based drug calculation sheet, trainees reported that it took longer to find the right dose since that form contained multiple other drugs. One trainee suggested that because epinephrine is one of the drugs that is most commonly administered during resuscitation, the dosages for intravenous (IV) and endotracheal tube (ETT) administration should be listed on a laminated card and placed inside the NICU emergency tackle box. This tackle box is taken to all deliveries attended by a NICU nurse. The use of this card has allowed for more timely identification of the correct dose by weight and administration route, reducing time to administration (Figure 2).

The second LST identified concerned the performance of neonatal resuscitation and initiation of oxygen therapy. Throughout multiple simulated scenarios, trainees had difficulty in assessing the extent to which the simulated newborn required assistance with ventilation and displayed uncertainty as to the need for oxygen as guided by the Neonatal Resuscitation Program (NRP) algorithm. To assist trainees with timely evaluation and management, the simulation team placed laminated cards on the radiant warmers in Labor and Delivery that address the target hemoglobin oxygen saturation levels by minutes of age according to NRP guidelines [12]. Furthermore, cards depicting the mnemonic MRSOPA (used to indicate the six steps recommended to address inadequate ventilation) were placed on the radiant warmer (Table 1). 

**Figure 3:**
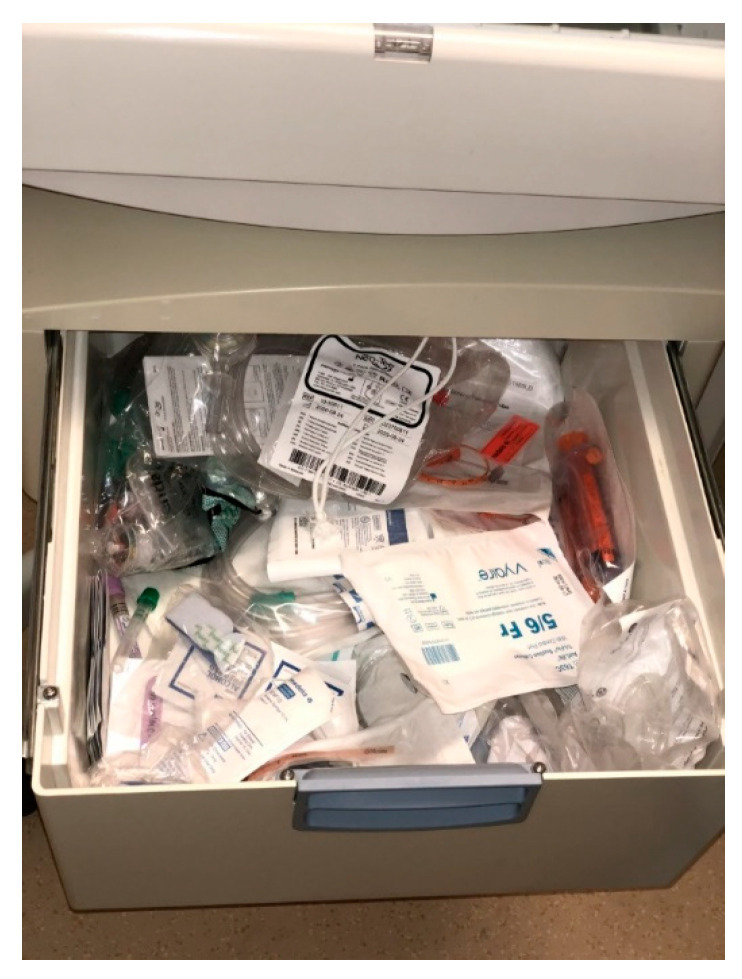
Mother–Baby Radiant Warmer Drawer Before Simulation.

The third LST identified pertained to the resuscitation equipment. One of our simulated scenarios involved the response to a newborn in the normal newborn nursery in the Mother–Baby Unit who became dusky during a lab draw. The newborn needed to be moved from a crib to the radiant warmer for resuscitation. Trainees participating in the scenario noted that the equipment and supplies that were needed to treat the simulated newborn were scattered haphazardly in the drawer of the radiant warmer, making it difficult to find them in a timely manner and potentially producing a negative impact on neonates requiring resuscitation (Figure 3). 

**Figure 4:**
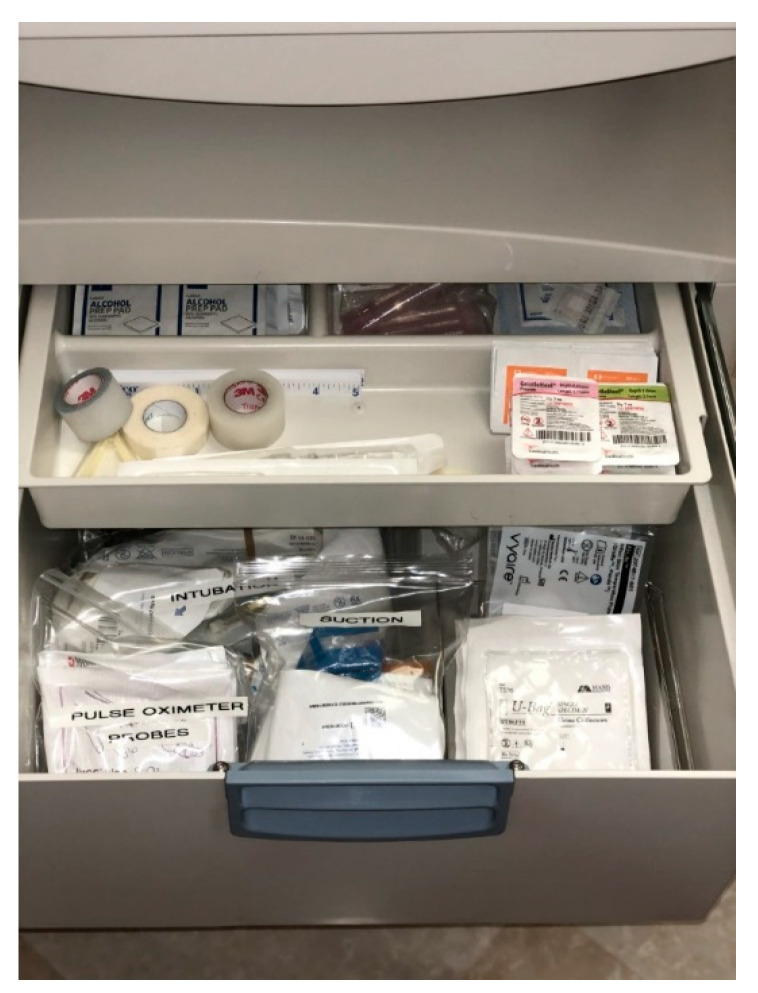
Mother–Baby Radiant Warmer Drawer After Simulation.

**Figure 5:**
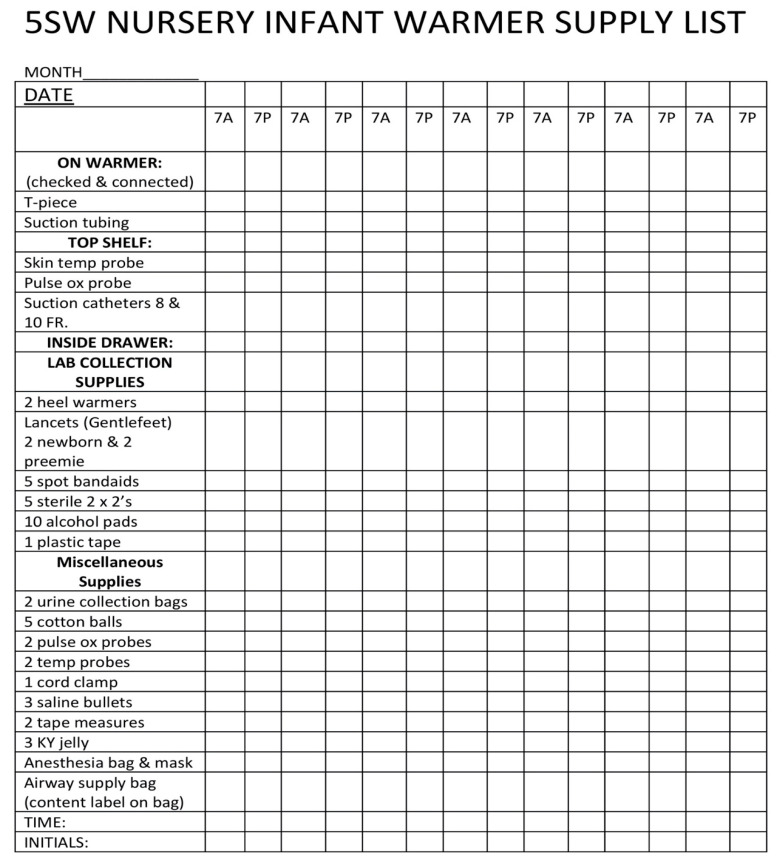
Mother–Baby Radiant Warmer Checklist.

As a result, the NICU team assisted the clinical coordinators in that unit with reorganizing supplies and equipment and developing a checklist to ensure that everything needed to resuscitate a newborn is present (Figure 4 and Figure 5).

The fourth LST involved the process of stabilizing a newborn with gastroschisis. This scenario truly reflects a high-risk, low-volume situation for our teams as a newborn with gastroschisis has not delivered at our hospital in more than five years and few of our staff have practical hands-on experience in managing a newborn with this condition. During the review of this recorded scenario, the CAPE team noted that other hospitals manage the stabilization of newborns with this congenital malformation in a different manner. This prompted us to contact the pediatric surgeons at the children’s hospital to which we typically transfer our surgical patients and inquire as to what procedure they prefer that we follow. This led to a change in our stabilization process to reflect these updated recommendations. We also modified our supplies and equipment based on that information and communicated these changes to all NICU staff via email, as well as during the staff huddles held prior to each shift. We also noted that our debriefings of this scenario provided an opportunity for staff to engage in rich discussions (essentially “debriefing themselves”); this activity has been shown to improve clinical reasoning that may translate to replicating debriefings in a real-life situation [13].

## 4. Discussion

While the *Simulating Success* QI project involved experienced neonatal healthcare professionals, much of the literature that describes debriefing, centers on training relatively inexperienced learners, not practicing healthcare professionals. Most authors convey the need for four phases of debriefing: reaction, description, analysis, and summary [14,15,16,17,18,19,20,21,22,23]. This is done primarily because of a belief that psychological distress is frequent during participation in simulated clinical events and, without first having an opportunity to “ventilate” pent up emotion, trainees being debriefed will not be able to participate effectively in a debriefing. Henricksen et al. examined the expression of psychological distress in 3900 subjects undergoing debriefing after simulated healthcare scenarios and found that <1% were perceived to manifest such distress [24]. Finally, patient outcome is not routinely emphasized and some authors state that discussion of patient outcome should be avoided, especially when outcome is poor [25]. These aspects of debriefing in healthcare conflate the difference between a technical performance debriefing (used to critique human and system performance) and a critical incident stress debriefing (conducted to provide psychological support after an emotionally and/or psychologically challenging event). A fundamental difference with CAPE’s Guiding Principles for Healthcare Debriefings [26] emphasizes that learning is better achieved through facilitated trainee discussions rather than didactic teaching provided by the facilitator. This facilitated discussion can be best achieved using a series of four “drill-down” questions [26]. CAPE’s debriefing principles also guide trainees to develop approaches to replicate actions that strengthen human and system performance and avoid activities that are ineffective or harmful [26]. Integrating CAPE’s Guiding Principles for Healthcare Debriefings was a change from our unit’s previous style of debriefing. There was a learning curve for the core team members to adapt and incorporate various elements of CAPE’s debriefing principles. Additionally, participants commented on the differences they experienced themselves and that utilizing CAPE’s debriefing principles opened more opportunities for discussion and active learning.

CPQCC’s *Simulating Success* collaborative was designed to assist participating NICUs implement a patient safety neonatal simulation and debriefing program. In the context of this QI collaborative, our community NICU benefitted from the expertise of CAPE and CPQCC faculty, and the QI specialist who helped critique our processes and support our goals. At its foundation, CPQCC utilizes the Institute for Healthcare Improvement (IHI) improvement framework—the Model for Improvement—which focuses on three questions and conducting Plan-Do-Study-Act (PDSA) cycles during the implementation phase of a collaborative [10]. The three questions are: What are we trying to accomplish? How will we know that a change is an improvement? What change can we make that will result in improvement? To address these questions, we conducted PDSA cycles that developed the change (plan), implemented the change (do), evaluated the change (study), and determined whether any modifications or revisions were needed (act) [27]. To answer the IHI’s three questions:

1. What are we trying to accomplish? *We sought to implement a simulation and debriefing program*.
2. How will we know that a change is an improvement? *We aimed to conduct two simulations and debriefings monthly and achieve an 80% participation rate by our nursing staff in two simulations during the yearlong implementation phase*.
3. What change can we make that will result in improvement? *We sought to identify LSTs, make changes to various resuscitation processes*, *and train additional team members in order to sustain the simulation and debriefing program*.

It is worthwhile to note that, from a QI collaborative perspective, a unit’s readiness for change is assessed before embarking in a QI collaborative. This assessment of the readiness for change can be evaluated by various unit and/or organizational context factors. Relevant stakeholders who can effect change, especially from a financial and human resource standpoint, should assess and understand the unit’s culture, leadership and financial support, and evaluation capabilities [28]. We have been involved with past CPQCC collaboratives; however, those collaboratives focused on reducing variation in the performance of healthcare professionals and/or standardizing changes in practice (e.g., antibiotic stewardship and increasing the frequency of breastmilk feedings). With *Simulating Success,* our goal was to implement and develop processes to sustain a simulation and debriefing program. To achieve this goal, we needed to ensure the significant financial resources necessary to support personnel, assistance from information technology services, allocation of sufficient time to allow core team members, nurses and respiratory therapists to participate in the simulated clinical scenarios. We were fortunate that our hospital’s nursing, respiratory, and physician leadership recognized the importance of a simulation and debriefing program to neonatal care. This support facilitated buy-in with the program by our staff.

Undertaking a QI project also means having the ability to collect and evaluate data, and then provide feedback to team members to advance QI improvements. While we acknowledge that our project’s Aim statements did not focus on patient outcome data, the LSTs identified by the trainees and the resultant process improvements did act to foster staff buy-in. We cannot underscore the importance of evaluation and feedback in quality improvement work—they are the foundations of “study” and “act” in PDSA cycles.

### Implementation Barriers

One of the concerns of the core team involved videotaping staff during the simulation and debriefing. In the past, videotaping simulated scenarios had not been a positive experience, as expressed by many NICU staff. We used a handheld iPad for recording and moved it around to attempt to capture the best views; this served to constantly remind staff that they were being filmed. The staff also expressed concern that mistakes made during simulation would be highlighted during playback during debriefings. For all these reasons, we had ceased recording simulated scenarios. At the beginning of *Simulating Success,* the core team was concerned that the videotaping and playback could make staff resistant to participating in this QI collaborative. These issues were addressed with staff members by the core team via one-on-one discussions and presentations to groups of professionals working in the NICU and Labor and Delivery. The core team assured staff that recordings would be used only as a debriefing tool to enhance training and not as a formal critique of their abilities. We also emphasized that our core team’s debriefing technique was backed by CPQCC and CAPE as a recommended technique. Nursing Leadership also approved the purchase of a “GoPro” camera (which has a small physical footprint) making recording less noticeable to the staff. As a result, many staff commented that they actually forgot that they were being videotaped during the scenarios. As the months progressed, the staff learned to “debrief themselves” as they watched the playback and became comfortable with CAPE’s debriefing methodology.

Another barrier was the challenge of a limited number of staff present during the simulations and the inability to bring on-duty NICU staff to Labor and Delivery for the simulated scenarios. We therefore utilized areas in the NICU such as a back corner or the isolation room and simulated as if we were in the labor and delivery area. We also ensured that each simulation and debriefing was completed in 30 min or less at the end of each shift. This allowed staff to complete all or most of their work prior to participating.

As with any QI project, there were barriers to participation. We, therefore, established that unit and organizational stakeholders shared our mental model as to how the program should proceed prior to its start. This shared mental model involved various context factors, such as financial and staff resources, in order to effect change and provide overall accountability for the implementation process and simulation program. To help with the program’s sustainability, we also actively engaged and started initial training with other interprofessional team members.

## 5. Conclusions

This manuscript describes our unit’s experience with implementing a simulation and debriefing program in a NICU QI collaborative setting. Our overall objective was to integrate an in-situ simulation and debriefing program in the context of our local unit and achieve widespread interprofessional simulation and debriefing experiences. Once we achieve consistent and sustained in-situ simulations and debriefings (conducting two simulations monthly for six months), our future goals include: (1) integrating CAPE’s debriefing techniques into debriefings following actual clinical events; (2) continuing to train the current members of our simulation team and recruit new members; and (3) using patient outcome data from the CPQCC database to inform future QI projects, using simulation as a method for evaluation and feedback.

## Figures and Tables

**Table 1:** The Steps of MRSOPA (modified from the Textbook of Neonatal Resuscitation [12]).

MRSOPA	ACTION
**M:**	adjust **M**ask
**R:**	**R**eposition mask
**S:**	**S**uction mouth and nose
**O:**	**O**pen mouth
**P:**	increase **P**ressure
**A:**	**A**lternate airway

## References

[1] Institute of Medicine (1999). To Err is Human.

[2] James J. (2013). A new, evidence-based estimate of patient harms associated with hospital care. J. Patient Saf..

[3] Wyckoff M.H., Aziz K., Escobedo M.B., Kapadia V.S., Kattwinkel J., Perlman J.M., Simon W.M., Weiner G.M., Zaichkin J.G. (2015). Part 13: Neonatal resuscitation: American Heart Association guidelines update for cardiopulmonary resuscitation and emergency cardiovascular care. Circulation.

[4] Shepherd C.K., McCunnis M., Brown L., Hair M. (2010). Investigating the use of simulation as a teaching strategy. Nurs. Stand..

[5] Rubio-Gurung S., Putet G., Touzet S., Gauthier-Moulinier H., Jordan I., Beissel A., Labaune J.-M., Blanc S., Amamra N., Balandras C. (2014). In situ simulation training for neonatal resuscitation: An RCT. Pediatrics.

[6] Lamberta M., Aghera A. (2020). Latent Safety Threat Identification via Medical Simulation. StatPearls [Internet].

[7] Wetzel E.A., Lang T.R., Pendergrass T.L., Taylor R.G., Geis G.L. (2013). Identification of latent safety threats using high-fidelity simulation-based training with multidisciplinary neonatology teams. Jt. Comm. J. Qual. Patient Saf..

[8] Couto T.B., Barreto J.K.S., Marcon F.C., Mafra A.C.C.N., Accorsi T.A.D. (2018). Detecting latent safety threats in an interprofessional training that combines in situ simulation with task training in an emergency department. Adv. Simul..

[9] Knight P., MacGloin H., Lane M., Lofton L., Desai A., Haxby E., Burmester M., Macrae D., Korb C., Mortimer P. (2017). Mitigating latent threats identified through embedded in situ simulation program and their comparison to patient safety incidents: A retrospective review. Front. Pediatr..

[10] Pai V.V., Lee H.C., Profit J. (2018). Improving uptake of key perinatal interventions using statewide quality collaboratives. Clin. Perinatol..

[11] Wells S., Tamir O., Gray J., Naidoo D., Bekhit M., Goldmann D. (2018). Are quality improvement collaboratives effective? A systematic review. BMJ Qual. Saf..

[12] Weiner G.M., Zaichkin J., American Academy of Pediatrics and American Heart Association (2016). Textbook of Neonatal Resuscitation (NRP).

[13] Bae J., Lee J., Jang Y., Lee Y. (2019). Development of simulation education debriefing protocol with faculty guide for enhancement clinical reasoning. BMC Med. Educ..

[14] Jaye P., Thomas L., Reedy G. (2015). ‘The Diamond’: A structure for simulation debrief. Clin Teach..

[15] Garden A.L., Le Fevre D.M., Waddington H.L., Weller J.M. (2015). Debriefing after simulation-based non-technical skill training in healthcare: A systematic review of effective practice. Anaesth. Intensiv. Care.

[16] Kolbe M., Weiss M., Grote G., Knauth A., Dambach M., Spahn D.R., Grande B. (2013). TeamGAINS: A tool for structured debriefings for simulation-based team trainings. BMJ Qual. Saf..

[17] Cheng A., Palaganas J., Eppich W., Rudolph J., Robinson T., Grant V. (2015). Co-debriefing for simulation-based education: A primer for facilitators. Simul. Healthc..

[18] Dreifuerst K.T. (2009). The essentials of debriefing in simulation learning: A concept analysis. Nurs. Educ. Perspect..

[19] Dreifuerst K.T. (2012). Using debriefing for meaningful learning to foster development of clinical reasoning in simulation. J. Nurs. Educ..

[20] Zigmont J.J., Kappus L.J., Sudikoff S.N. (2011). The 3D model of debriefing: Defusing, discovering, and deepening. Semin. Perinatol..

[21] Eppich W., Cheng A. (2015). Promoting Excellence and Reflective Learning in Simulation (PEARLS): Development and rationale for a blended approach to health care simulation debriefing. Simul. Healthc..

[22] Gardner R. (2013). Introduction to debriefing. Semin. Perinatol..

[23] Rudolph J.W., Simon R., Raemer D.B., Eppich W.J. (2008). Debriefing as formative assessment: Closing performance gaps in medical education. Acad. Emerg. Med..

[24] Henricksen J.W., Altenburg C., Reeder R.W. (2017). Operationalizing healthcare simulation psychological safety: A descriptive analysis of an intervention. Simul. Healthc..

[25] Lyons R., Lazzara E.H., Benishek L.E., Zajac S., Gregory M., Sonesh S.C., Salas E. (2015). Enhancing the effectiveness of team debriefings in medical simulation: More best practices. Jt. Comm. J. Qual. Patient Saf..

[26] Halamek L.P., Cady R., Sterling M.R. (2019). Using briefing, simulation and debriefing to improve human and system performance. Semin. Perinatol..

[27] Institute for Healthcare Improvement Plan-Do-Study-Act (PDSA) Worksheet. http://www.ihi.org/resources/Pages/Tools/PlanDoStudyActWorksheet.aspx#:~:text=The%20PDSA%20cycle%20is%20shorthand,to%20the%20test%20(Act).

[28] Stetler C.B., Damschroder L.J., Helfrich C.D., Hagedorn H.J. (2011). A guide for applying a revised version of the PARIHS framework for implementation. Implement. Sci..

